# Von Willebrand factor targeted thrombolysis in canine basilar artery occlusion

**DOI:** 10.3389/fneur.2024.1436291

**Published:** 2024-10-09

**Authors:** Arianna Carfora, Blake Holthaus, Simon Yacoub, Dominic Franceschelli, Matthew Joseph, Michael W. Milks, Ian Mandybur, Cole Anderson, Catherine Lee, Allyson Huttinger, Mohammad Shujaat, Debra G. Wheeler, Bruce Sullenger, Shahid M. Nimjee

**Affiliations:** ^1^Department of Neurological Surgery, The Ohio State University Wexner Medical Center, Columbus, OH, United States; ^2^Department of Internal Medicine, The Ohio State University Wexner Medical Center, Columbus, OH, United States; ^3^Department of Radiology, The Ohio State University Wexner Medical Center, Columbus, OH, United States; ^4^Duke Translational Research Institute, Duke University, Raleigh Durham, NC, United States

**Keywords:** acute ischemic stroke, posterior circulation, thrombolysis, von Willebrand factor, aptamer

## Abstract

**Background and purpose:**

Posterior circulation strokes, accounting for 20% of acute ischemic strokes, significantly contribute to morbidity and mortality. Fibrinolysis by rtPA improves outcomes in stroke but the risk of intracranial hemorrhage limits benefit. Arterial recanalization of basilar artery occlusion by thrombolysis or endovascular thrombectomy improves outcomes in posterior circulation strokes. This study investigates a VWF-targeting RNA aptamer as a safer and more effective alternative to rtPA in a canine model.

**Materials and methods:**

Autologous clots were placed into the basilar artery to induce stroke in 24 beagles. To compare reperfusion, 0.9 mg/kg rtPA, 0.5 mg/kg BB-031, or vehicle were administered 60 min after the initiation of occlusion. Digital subtraction angiography, laser speckle imaging and magnetic resonance imaging were used to assess recanalization, reperfusion and infarct volume, respectively.

**Results:**

Treatment with BB-031 resulted in recanalization of the posterior circulation on digital subtraction angiography with no evidence of microembolism assessed at sacrifice. 66.5% of animals treated with BB-031 resulted in reperfusion with a TICI score of ≥1 whereas vehicle remained at TICI score 0 as did all but one rtPA animal at sacrifice. Improved perfusion was seen in the basilar artery and surrounding blood vessels visualized through the cranial window with laser speckle imaging to ~47% of its original baseline in BB-031 group compared to rtPA at 37% and vehicle at 22%. Finally, BB-031-treatment resulted in an approximate 32% mean infarct volume, significantly smaller on magnetic resonance imaging compared to 56% in vehicle treated and 48% with rtPA treatment.

**Conclusion:**

Targeted inhibition of VWF by BB-031 increased recanalization and reperfusion, and reduced infarct volume in a canine model of BAO stroke. It represents a promising target based on preliminary results for treating acute ischemic stroke.

## Introduction

Acute ischemic stroke (AIS) is a leading cause of death, with 3.5 million worldwide yearly (AHA Stats Tsao 2022). Intravenous recombinant tissue plasminogen activator (rtPA) administered within 4.5 h of symptom onset is standardized care for AIS, but thrombolysis is unreliable, and complete recanalization rates are low. Unfortunately, rtPA treatment induces symptomatic intracranial hemorrhage in up to 6% of patients, resulting in significant morbidity and mortality, in part because there is no way to reverse rtPA activity. Although basilar artery occlusion (BAO) represents a small percentage of AIS (~25%), it is by far the most devastating, with high mortality of ~85–95% even with current anticoagulant or fibrinolytic agents and severe disability in those that survive the initial insult (1–4). Embolism or thrombosis cause most vertebrobasilar strokes to the vertebral or basilar arteries (BA), with ~35% caused by thrombosis in the middle or distal basilar artery. Still, the BA is the main vessel supplying the brainstem, occipital lobes, part of the cerebellum, & the thalami. BAO presents with a range of sequela, including hemiplegia to coma resulting in death, although the risk of posterior vs. anterior strokes is about half (2, 3, 5). The location of the BA in the smaller area of the posterior circulation in the brainstem and cerebellum containing neural tracts, which support life, makes bleeding, infarction, and edema in this region a more critical and urgent need for recanalization (1). BAO is also associated with a significantly higher risk of recurrent strokes in the first few weeks (2) and slower recovery, commonly still improving even after 3 months due to lower blood flow velocities during ischemia compared to the middle cerebral artery (MCA), the site of the majority of anterior occlusions (1, 3). Mechanical thrombectomy (MT) quickly and efficiently induces recanalization at TICI 2b/3 in 90.3% of BAO patients. Still, it comes with a high complication rate compared to anterior occlusion strokes and necessitates interventional expertise that is not available uniformly. Posterior circulation strokes come with a longer time lapse before treatment & longer duration of procedures to attempt recanalization, resulting in lower reperfusion rates (6).

Current thrombolytics in BAO result in low or moderate reperfusion, regardless of whether the administration is intravenous (IVT) or intra-arterial (IAT) (3, 5), but do result in a lower rate of hemorrhagic complications compared to anterior occlusions. Most importantly, IVT can be applied without delay after BAO diagnosis & may help reflow at the BA and the capillary level while endovascular interventions, while effective, are limited to hospitals that perform this procedure (7). Therefore, a critical need exists to investigate new targets that effectively and rapidly lyse posterior clots, can be reversed to negate the risk of hemorrhage, provide a safety profile necessary for use, and lyse distal embolism. Moreover, an ideal therapy must be available in all medical centers and not reliant on transportation to specialized urban centers.

von Willebrand factor (VWF) is an important blood homeostasis glycoprotein that forms a bridge with damaged endothelium by binding to platelet GP1bα. Under normal conditions, the binding site for GP1bα in the A1 domain of VWF is not accessible while circulating, but during high shear as in AIS, VWF binds collagen to the exposed extracellular matrix (ECM), which is integral in platelet adhesion, aggregation, and thrombus formation. VWF is produced exclusively by megakaryocytes and endothelial cells and directly secreted or stored in Weibel Palade bodies of the endothelium or in platelet *α*-granules. During vascular injury, VWF secretion increases in the form of ultra large multimers (UL-VWF), which will be rapidly cleaved by A Disintegrin And Metalloprotease ThromboSpondin motif repeats, number 13 (ADAMTS13) to less hyperactive fragments until ADAMTS13 becomes deficient in the circulation. VWF not only plays a role in arterial and venous thrombosis, but in atherosclerosis, and thrombosis accompanying malaria, sepsis, and sickle cell anemia, where ADAMTS13 activity is at or near normal levels. Therefore, ADAMTS13 cleavage is not the only mechanism that can regulate VWF adhesion and cleavage (8). UL-VWF does not require an agonist to induce thrombosis, but will spontaneously bind platelets and exacerbate aggregation. Multiple studies conclude that high VWF circulating levels drive poor outcomes in stroke patients (8–12). On the other hand, patients with Type 1 von Willebrand Disease with ~70% quantitative deficiency of VWF are protected from cardiovascular thrombotic disorders (13).

Aptamers are a class of deoxy-ribonucleic (DNA) or ribonucleic acid (RNA) ligands that fold into 3D structures, binding to and inhibiting the function of their target proteins with high affinity and specificity (14). Aptamer advantages include the ability to modify their half-life, unlimited shelf life, low to no immunogenicity and most importantly, their activity can be rapidly reversed. Aptamer reversal is achieved by designing a second molecule with a homologous sequence to the aptamer. Our initial research generated the first antidote-mediated regulation of a molecule and produced the first rationally designed reversible anticoagulant (14–20). In the setting of anti-thrombotic therapy, reversal of the active drug is essential to minimizing morbidity and mortality associated with hemorrhage. Prior attempts to develop potent antiplatelet agents targeting VWF with aptamers have fallen short because of the inability to reverse drug activity when it is no longer needed (18, 19, 21, 22, 23). We developed, truncated, and chemically modified an RNA aptamer, BB-031, which binds to VWF with high affinity and specificity and created a reversal oligonucleotide, BB-025, which inactivates BB-031 within minutes (24). BB-031 inhibited platelet adhesion under high shear stress in a dose-dependent manner and prevented platelet aggregation. These findings were corroborated in a large animal model of canine carotid thrombosis. They showed that aptamer-mediated recanalization of the carotid artery not only restores blood flow but accomplishes this without inducing intracranial hemorrhage or shedding of embolic clots to the brain (25).

Targeting VWF has distinct advantages over current AIS anti-platelet and anti-thrombotic, particularly concerning the severe subset of basilar artery occlusion. As summarized in the extensive review by Rana et.al, acute AIS treatments suffer from the risk of uncontrollable bleeding because they target physiological platelet response to elicit thrombosis but also disturb normal blood homeostasis (26). VWF protects FVIII from proteolytic inactivation and in concert, FVIII and VWF maintain homeostasis after endothelial injury. Low VWF levels accompany low FVIII levels, exacerbating bleeding. Only treatments which sustain VWF and FVIII and thus homeostasis, will negate this response, of which there are currently none which have proven efficacious clinically in AIS. In addition, inflammation pertaining to BAO is an area where treatment selection can improve long term outcome due to the longer recovery time. Reduced synthesis and increased cleavage by thrombin sets up an unbalanced milieu whereas ADAMTS13 activity is impaired, building up hyperactive UL-VWF. Potentiation of inflammation without replacement of ADAMTS13 or inhibition of VWF results in UL-VWF strings with P-selectin promoting platelet aggregation and additional leukocyte recruitment. Unfortunately, current anti-platelet drugs are minimally effective under the double scenario in AIS of high shear stress and high VWF levels. Large scale studies to compare the VWF levels between BAO and MCAO have not been completed to determine whether VWF levels after BAO, whether circulating or at the site of thrombosis, are more indicative of resistance to current treatment. Mortality of BAO patients at ~80–90% without recanalization is unacceptable. In the current investigation, we examined the thrombolytic efficacy and safety of BB-031 compared to rtPA in a preclinical model of nonsurvival basilar artery occlusion.

## Methods

### Experimental parameters

#### Animals

Twenty-four adult male and female beagles (Covance, Denver, PA) were used to complete this study with a mean age of 17.4 months and mean weight of 9.9 kg (Table 1). All procedures were approved by the Institutional Animal Care and Use Committee at The Ohio State University and were performed in compliance with the Animal Welfare Act and the Guide for Care and Use of Laboratory Animals (NRC 2011). Feeding was restricted to 12 h before anesthesia was induced. Analgesia was not provided to canines as procedures maintained deep anesthesia through time of sacrifice. Investigations were concluded in each canine group when significance was reached with n numbers to minimize the number of canines used.

**Table 1:** Baseline canine physiological data between treatment groups collected day of basilar artery occlusion.

Parameter	Vehicle (*n* = 8)	rtPA (*n* = 7)	BB-031 (*n* = 9)	*p*-value		
				Vehicle vs. rtPA	Vehicle vs. BB-031	rtPA vs. BB-031
Sex (M/F)	3/5	3/4	3/6			
Weight (kg)	10.6 ± 1.6	9.7 ± 1.1	9.5 ± 1.1	0.233	0.116	0.724
Age (weeks)	17.4 ± 4.3	17.2 ± 4.0	17.5 ± 3.6	0.927	0.959	0.877
Heart rate (bpm)	91 ± 17	85 ± 9	89 ± 4	0.419	0.735	0.251
PFA (seconds)	62 ± 11	62 ± 19	54 ± 7	0.990	0.090	0.259
Systolic BP (mmHg)	84 ± 12	86 ± 12	82 ± 10	0.752	0.713	0.478
Diastolic BP (mmHg)	52 ± 8	51 ± 8	48 ± 5	0.812	0.229	0.372
Mean BP (mmHg)	64 ± 10	62 ± 9	60 ± 7	0.692	0.349	0.624
LSI perfusion (pu)	188 ± 60	195 ± 30	212 ± 22	0.791	0.293	0.225
VWF (ng/mL)	4.03 ± 5.55	7.87 ± 7.17	8.04 ± 5.41	0.867	0.794	0.618
ADAMTS13 (ng/mL)	4.03 ± 4.85	5.12 ± 3.61	6.69 ± 2.49	0.560	0.978	0.676

#### Intravenous treatment

Sixty minutes after initiation of BAO, we injected a 10 mL bolus of vehicle (PBS, pH 7.4), a 10 mL bolus of 0.5 mg/kg BB-031 *or* 10% 0.9 mg/kg rtPA bolus (Alteplase, Genentech, San Francisco, CA) (Table 1 Timeline) over 5 min followed by an additional 40 min infusion at 2 mL/min utilizing a timed perfusion pump (PHD 2000 Infusion, Harvard Apparatus, Holliston, MA) to replicate clinical rtPA administration (27–29). The remaining infusion for vehicle and BB-031 groups after initial bolus was PBS, pH 7.4. BB-031 was synthesized by the same company as all previous animal experiments per identical Principal Investigator sequence instructions, HPLC purified, desalted and lyophilized as a sodium salt (Nitto Denko Avecia Inc. Milford, MA). Upon obtaining animal weight day of surgery, BB-031 was dissolved in PBS, concentration quantified by spectrophotometry and administered. rtPA was dissolved in PBS, pH 7.4, immediately before use.

#### Canine model of thromboembolic BA occlusion

**Figure 1:**
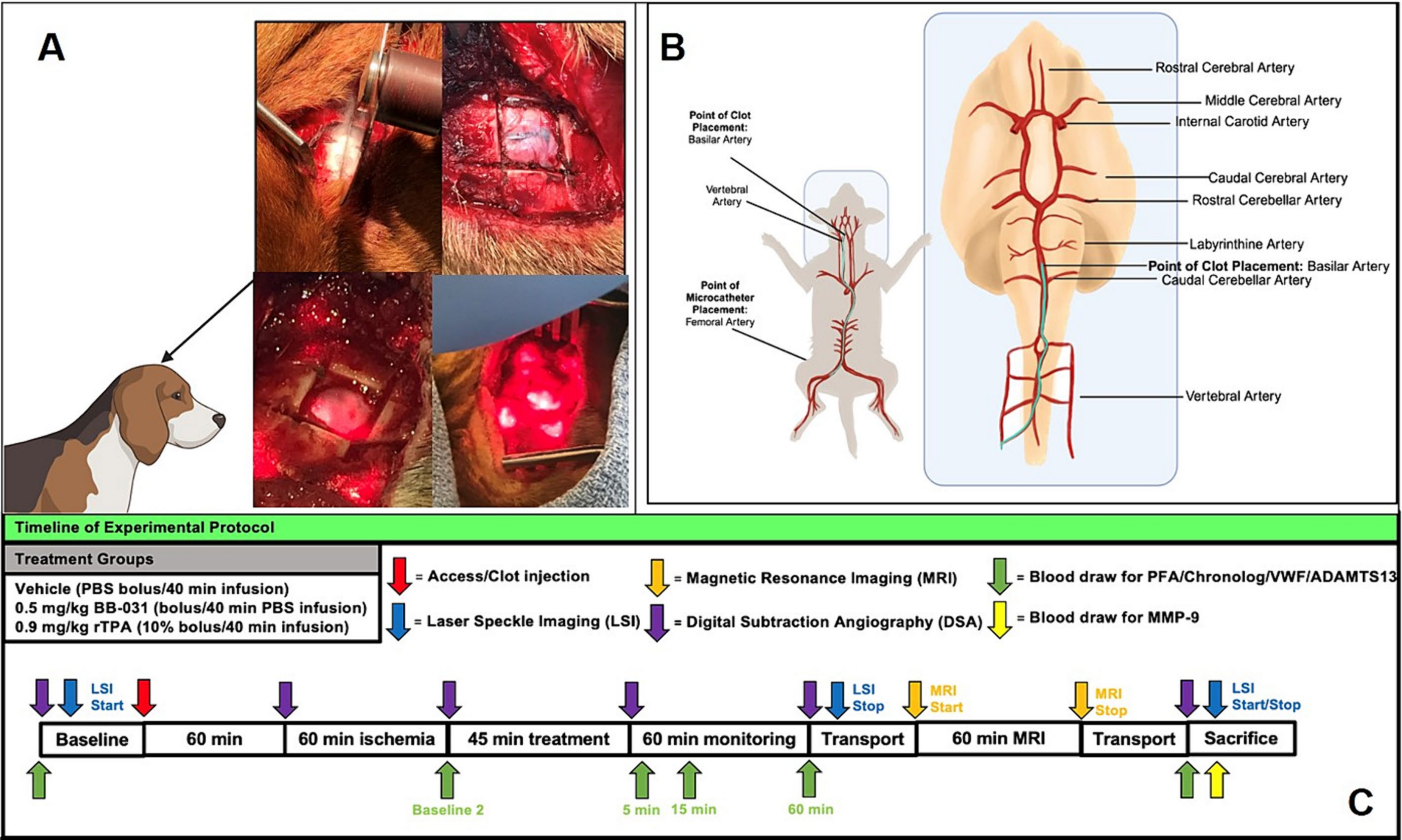
Experimental protocol for basilar artery occlusion and treatment with corresponding time points of laser speckle imaging (LSI), magnetic resonance imaging (MRI) and whole blood draws. **(A)** Creation of cranial window for laser speckle imaging in upper panels and bone removed and laser positioning during scanning in the bottom panels. **(B)** Femoral artery access and microcatheter path to basilar artery with clot placement location. **(C)** Treatment groups and timeline for all data collection of LSI, MRI, surgical procedure, digital subtraction angiography, and blood draw for protein analysis.

Thromboembolic BA occlusion was induced as previously published (30). Briefly, animals received 0.2 mg/kg intramuscular acepromazine before intubation. A 20-gauge catheter was placed in the cephalic vein, and anesthesia was induced with intravenous administration of 10 mg/kg ketamine and 0.025 mg/kg midazolam. Hounds were intubated and mechanically ventilated at 12 respirations per minute with constant flow anesthesia (2–3% isoflurane), and vitals were continuously monitored. The equipment that recorded canine vitals was mobile with battery back-up (Passport, Datascope) and followed the animal during transport to and from MRI and long monitoring leads connected to the canines inside the magnetic resonance (MR) imager. A 1.5 cm^2^ craniotomy window was cut with a bone saw to provide access for laser speckle imaging (LSI) (Figure 1A). Animals were placed supine with the head slightly extended in an inflated MRI compatible Hug-u-Vac (Surgical Positioning Systems, Sports Doc Inc., Salem, OR) which maintained body temperature at 37 ± 1°C recorded with a rectal probe throughout the entire procedure. A cutdown was performed over the right femoral artery to insert a 7F arterial sheath via Seldinger technique. A 16-gauge angiocatheter was inserted into the right femoral vein to extract blood for autologous clot preparation, blood gas analysis (BGA), and complete blood count (CBC). To create the autologous clot, 5 mL of whole blood was drawn into a 5 mL BD Luer lock syringe and added to 0.5 g of barium sulfate (Ba_2_SO_4_) in a plastic serum blood collection tube with rolling for 30 s then sat undisturbed for 60 min before injection at room temperature (31). Sixty minutes allows for natural clot retraction and more stable occlusion and in the event target vessel access took longer than 60 min, the clot preparation was repeated. To begin recording baseline digital subtraction angiography (DSA) before accessing the middle basilar artery, a 4F guiding catheter was advanced under fluoroscopic guidance, using a retrograde trans-aortic approach, into the 7F arterial sheath previously placed into the right femoral artery through a vertebral artery to the base of the basilar artery (Figure 1B). Two milliliters of contrast agent with normal saline allowed the identification of the canine vasculature (OEC 9800 Cardiac Plus). Heart rate (HR) and blood pressure (BP) were monitored invasively through the arterial sheath throughout the procedures, and CBC and BGA were completed at baseline before BAO and at the time of sacrifice as previously published in the same model (32).

After 60 min, the autologous clot was cut into small pieces with both fibrin-rich and erythrocyte-rich layers, loaded into a high-pressure syringe (Medallion, Merit Medical, South Jordan, UT), injected through the microcatheter into the *middle* of the BA, stabilized for 3 min, and followed by 1 mL of contrast (350 mg/I, Omnipaque, GE) to flush the remaining clot from the catheter. Injection stopped when the vessel was occluded, verified by additional fluoroscopy with contrast that did not pass the occlusion into the distal BA and precipitous decrease in cerebral perfusion by laser speckle imaging. This process, including clot injection, contrast, and verification of occlusion, was performed in approximately 5 min. Clot placement and complete basilar artery occlusion were confirmed from DSA with Thrombolysis in the Cerebral Infarction scale (TICI) 0 flow, and BAO start time was recorded. After 60 min of ischemia, treatments were infused intravenously, followed by 60 min of monitoring before transport for MRI, a short 2–3 min drive from the angiography suite. The arterial sheath was left in place for additional anesthesia when necessary. Animals were sacrificed upon return to the angiography suite after completion of final DSA, and blood draws approximately 4 h after BAO initiation.

### Imaging modalities

#### Laser speckle imaging of blood flow

The high-resolution laser speckle imaging camera system (PeriMed Inc., Las Vegas, NV) was configured to focus the laser on the cranial window as previously described, and perfusion was recorded continuously except with interruptions while performing angiograms/MRI and transport to and from MRI (Figure 1A) (30). Briefly, data was acquired from a 1.5 cm x 1.5 cm field of view using a 785 nm wavelength and 80 mW lasers with a sampling rate of 60 Hz at a working distance of 10 cm. Images were recorded at 15 frames per second, and intensity and variance calculations were completed with spatiotemporal averaging over a 5  ×  5 pixel area with 5 frames. The overall frame rate for the intensity and variance data was 3 frames per second. Time-of-interest (TOI) was chosen from the real-time perfusion graphs to include lower peaks to exclude motion-related respiratory artifacts. Relative perfusion units were averaged over each 10-s interval using PimSoft v1.4 software. Raw data was converted into binary files and processed into meaningful images of the vasculature. The re-tooled LASCA algorithm (rt-LASCA) was used for the variance of the contrast data over time to determine the locations of vasculature as previously described (32).

#### Magnetic resonance imaging, infarct, and recanalization analysis

Anesthetized canines were placed supine, head-first in a Siemens Prisma 3 Tesla MRI 60 cm bore scanner and a 32-channel head coil with enhanced parallel imaging to obtain canine brain images as previously published (30, 33). Briefly, image sequences included diffusion-weighted imaging (DWI), with subsequently generated apparent diffusion (ADC) maps, T2-weighted, Fluid attenuated inversion recovery (FLAIR), and T2-weighted gradient echo images. The system utilized allows fast scanning in optimal spatial and temporal resolutions with 80 mT/m gradients to generate high-quality T2-weighted, Diffusion-weighted, and magnetic resonance angiography (MRA) images. Initially, localizer scans were performed to acquire pilot images before anatomical imaging. T2-weighted imaging was then performed (Parameters: FOV = 130 mm, Matrix size = 320 × 320, pixel size = 0.3 × 0.3 mm, Slice thickness = 3 mm, TR = 4 s, FA = 180 degrees, BW = 255 Hz/pixel, NEX = 2, TE = 75 ms, Resolution = 2.4615 pixels per mm), followed by FLAIR images for detection of edema. DWI sequences were used to detect acute ischemic strokes (Parameters: FOV = 149 mm × 149 mm, Matrix size = 132 × 100, pixel size = 0.3 × 0.3 mm, slice thickness = 4 mm, TR = 4.6 s, FA = 90 degrees, BW = 255 Hz/pixel, NEX = 1, TE = 86 ms, Resolution = 0.93 pixels per mm), confirmed by the generated ADC maps. DICOM images were transferred for post-processing, and infarct volumes were calculated using Horos open-source software based on OsiriX™. MRI analysis of infarct volume, hemorrhage, and edema was performed by a blinded board-certified neuroradiologist as previously described (30, 33). Hematoxylin and eosin (H&E), in addition to 2,3,5-triphenyl-2H-tetrazolium chloride (TTC) staining, were used as previously described to verify infarct size and identify hemorrhage in the brain sections affected by BA occlusion (25).

Thrombolysis in Cerebral Infarction scale (TICI) was assessed before and after clot placement, 60 min after BAO, 60 min after treatment, and before sacrifice. TICI scores were determined using previously established parameters (34).

### Platelet response to VWF inhibition

#### Analysis of aggregation status in canine blood

We collected and anticoagulated whole blood from the femoral artery for platelet and circulating protein assays from baseline to sacrifice at designated time points (Figure 1).

##### VWF/ADAMTS13/MMP-9

Whole blood was anticoagulated in sodium citrate vacutainers, platelet-rich plasma isolated, and VWF, ADAMTS13, and Matrix metalloproteinase-9 (MMP-9) measured using enzyme-linked immunosorbent assays (ELISA) which measure VWF antigen (VWF:Ag) and ADAMTS13 expressed as international units per deciliter (IU/dL) then graphed as ng/ml from standard curves (MyBioSource, Inc., San Diego, CA). MMP-9 was measured at baseline and at the time of sacrifice with a canine-specific ELISA kit from the same company.

##### Platelet function analysis

Whole blood was anticoagulated in lithium heparin vacutainers, and platelet function was measured utilizing a PFA-100 (Siemens Healthcare Diagnostics, Inc., Tarrytown, NY), a bench-top automated instrument that assesses primary platelet hemostasis under shear stress. One milliliter of whole blood was introduced into a disposable test cartridge that contained a membrane impregnated with collagen plus ADP, traveled through a 200 μM capillary opening, and was allowed to incubate for 5 min at 37°C. Platelet closing time, a maximum of 300 s, was recorded in duplicate for all samples and reported as a mean closing time.

##### Whole blood impedance aggregometry

Whole blood was anticoagulated in lithium heparin vacutainers, diluted 1:1 in 0.9% saline, and allowed to rest for 30 min at 37°C. Platelet aggregation was measured by the change in electrical impedance between two electrodes when agonist was induced (final concentration of 1 μg/μL botrocetin, 3.2 μg/μL collagen, and 20 μM ADP). After 6 min, the recording was stopped, and amplitude, slope, lag time, and area under the curve (AUC) were analyzed for each time point/agonist (Chronolog Model 300 Corporation, Havertown, PA). All agonists were purchased from Chronolog Corporation.

## Results

### Physiological response to thromboembolic BAO and treatments

There was no significant difference in baseline physiological between hounds in different treatment groups (*n* = 15 females, 9 males, Table 1). Female canines have been reported to have lower resting HR and BP, but we did not see a difference in this cohort of beagles (MAP in females 62 ± 8 vs. males 60 ± 6 mmHg, HR was 92 ± 9 bpm in females vs. 91 ± 13 in males). Although we saw an increase in heart rate from baseline to BAO through time of sacrifice, the increase was consistent across all treatment groups, as was the increase from baseline to BAO in MAP, SAP, and DAP, but did not reach significance and had decreased by time of treatment 60 min later. Parameters included in standard blood gas analysis (pH, pCO_2_, and pO_2_) at baseline for all hounds were not significantly different regardless of treatment group. They were still within the acceptable physiological range at the time of sacrifice provided by The Ohio State University Veterinary Medical College, who performed the analysis (data not shown). The number of platelets, red blood cells, and hemoglobin at the time of sacrifice were unremarkable with any treatment compared to baseline. White blood cells, not surprisingly as immune cell infiltration has been reported as early as 30 min after BAO, were significantly increased compared to baseline in every treatment group but, again, did not differ.

### Thrombolytic status in canine blood before and after BAO

#### Bleeding safety profile

We collected whole blood after anesthesia was induced, before any vascular manipulations (baseline), 60 min after occlusion (baseline 2, immediately before treatment), then 5, 15, and 60 min after treatment was completed, and lastly, at the time of sacrifice (~4 h after BAO). Isolated plasma was used to complete the measurement of circulating VWF, ADAMTS13, and MMP-9, while whole blood was used for Platelet Function Analysis (PFA) and Whole Blood Impedance Aggregometry (WBIA). Table 1 includes baseline values and number of animals/group.

##### VWF/ADAMTS13/MMP-9

**Figure 2:**
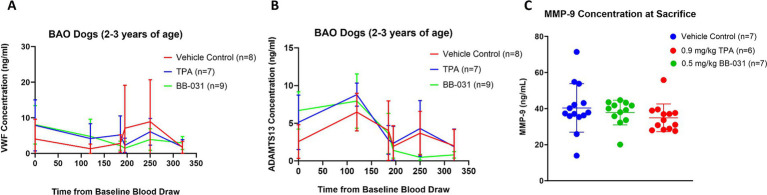
Protein changes with BAO and treatment. **(A)** von Willebrand Factor (VWF) decreased from baseline to time of BAO in all groups during access, catheter insertion and clot placement. 0.5 mg/kg BB-031 decreased with VWF inhibition while 0.9 mg/kg rtPA and vehicle further increased. There was no significance between groups ~4 h after BAO at time of sacrifice. **(B)** A Disintegrin And Metalloprotease ThromboSpondin motif repeats, number 13 (ADAMTS13) was not significantly different at baseline through 60 min after BAO with all groups except BB-031. All groups decreased with treatment, but only BB-031 decreased significantly beginning 250 min after baseline draw and maintained that decrease to time of sacrifice. **(C)** Matrix metalloproteinase-9 (MMP-9) decreased in both BB-031 and rtPA treatment groups but neared but did not reach significance.

There was no significant difference between groups in either VWF or ADAMTS13 levels at baseline or after 60 min of BAO (Figure 1). 0.5 mg/kg BB-031 treatment resulted in a large VWF *decrease* 5 min after administration (2.17 ± 1.28 ng/mL, *n* = 9), compared to an *increase* in rtPA (5.20 ± 2.33 ng/mL, *n* = 7), and vehicle (2.80 ± 1.43 ng/mL, *n* = 7), and the BB-031 group continued decreasing until the 60 min blood draws and was still only 36.5% of the original baseline mean (2.94 ± 1.70 ng/mL) at time of sacrifice (Figure 2) (*n* = 9). VWF levels in both vehicle and rtPA groups *increased* throughout the monitoring time after treatment, then vehicle decreased to 37.9% (1.53 ± 1.68 ng/mL) and rtPA to 18.0% (1.42 ± 2.00 ng/mL) of their original baseline.

Not surprisingly, with a decrease in VWF levels in all groups from baseline through basilar artery access and ischemia came a concurrent *increase* in ADAMTS13 levels to continually cleave the large multimeric VWF into a manageable size for further degradation. ADAMTS13 levels then decreased in all groups, beginning with blood draws 5 min after treatment and continuing to 60 min, where BB-031 (0.47 ± 0.39 ng/mL) further decreased compared to vehicle (3.68 ± 2.89 ng/mL, *p* = 0.005) and rtPA (4.30 ± 3.69 ng/mL, *p* = 0.008). There was no significant difference in MMP-9 levels with any treatment at baseline to the time of sacrifice (Figure 2).

##### Platelet function analysis

**Figure 3:**
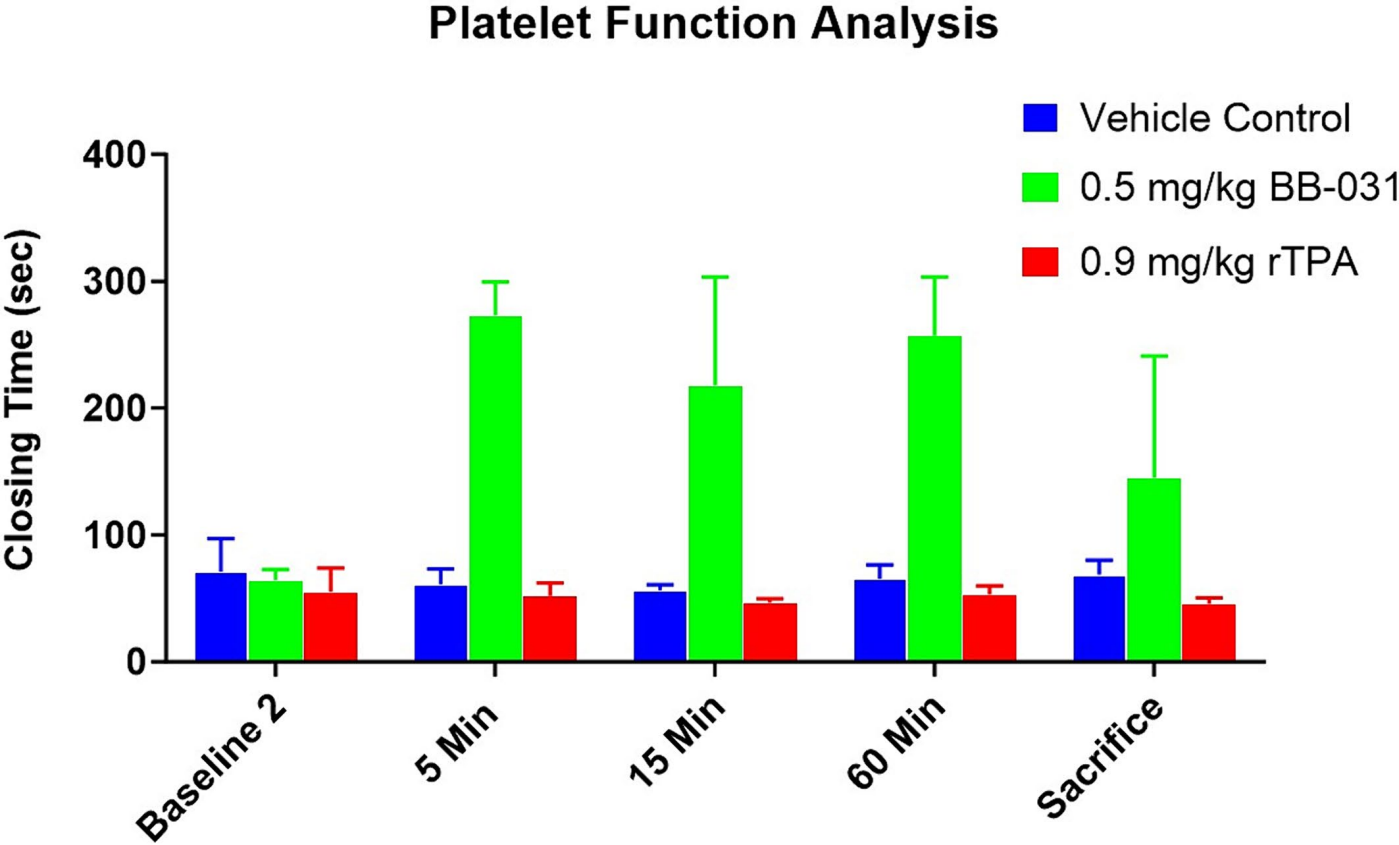
Platelet function analysis (PFA) changes with BAO and treatment. Although there was no difference from baseline with PFA closing time compared to baseline 2 (60 min after BAO but before treatment) in any treatment group, there was a precipitous increase in closing time with 0.5 mg/kg BB-031 at 5, 15, and 60 min after treatment was completed. Four hours after BAO, canines receiving BB-031 still had a mean closing time ~ 3 times greater than their mean baseline values (*n* = 6/group).

Although platelet closing times were not significantly different between canines *before* treatment (Table 1), 5 min after 0.5 mg/kg BB-031 treatment, mean closing time increased to 273 ± 26 s compared to 31 ± 11 s with vehicle (*p* < 0.0001) and 23 ± 10 s with rtPA (*p* < 0.0001) (Figure 3). At 15 min after treatment, mean closing time with BB-031 had decreased but was still significantly elevated (218 ± 85 s) compared to 25 ± 5 s with vehicle (*p* < 0.0001) and 21 ± 3 s with rtPA (*p* < 0.0001). Sixty minutes after intravenous treatment, the difference continued significantly with closing time for BB-031 at 257 ± 46 s compared to 29 ± 11 s with the vehicle and 24 ± 7 s with rtPA (*p* < 0.0001). Lastly, a full 140 min after treatment was completed, at the time of sacrifice, mean closing time for BB-031-treated canines was still significantly longer at 145 ± 96 s compared to 30 ± 11 s with vehicle (*p* = 0.022) and 20 ± 5 s with rtPA (*p* = 0.002) (Figure 3) more than 200% higher than the original baseline. Closure time depends on platelet count and hematocrit, but more importantly, VWF is essential for platelet plug formation under shear stress, which is simulated in this assay. Neither vehicle nor rtPA group’s mean closing time significantly deviated from their original baseline or each other throughout the experiment.

##### Whole blood impedance aggregometry

**Figure 4:**
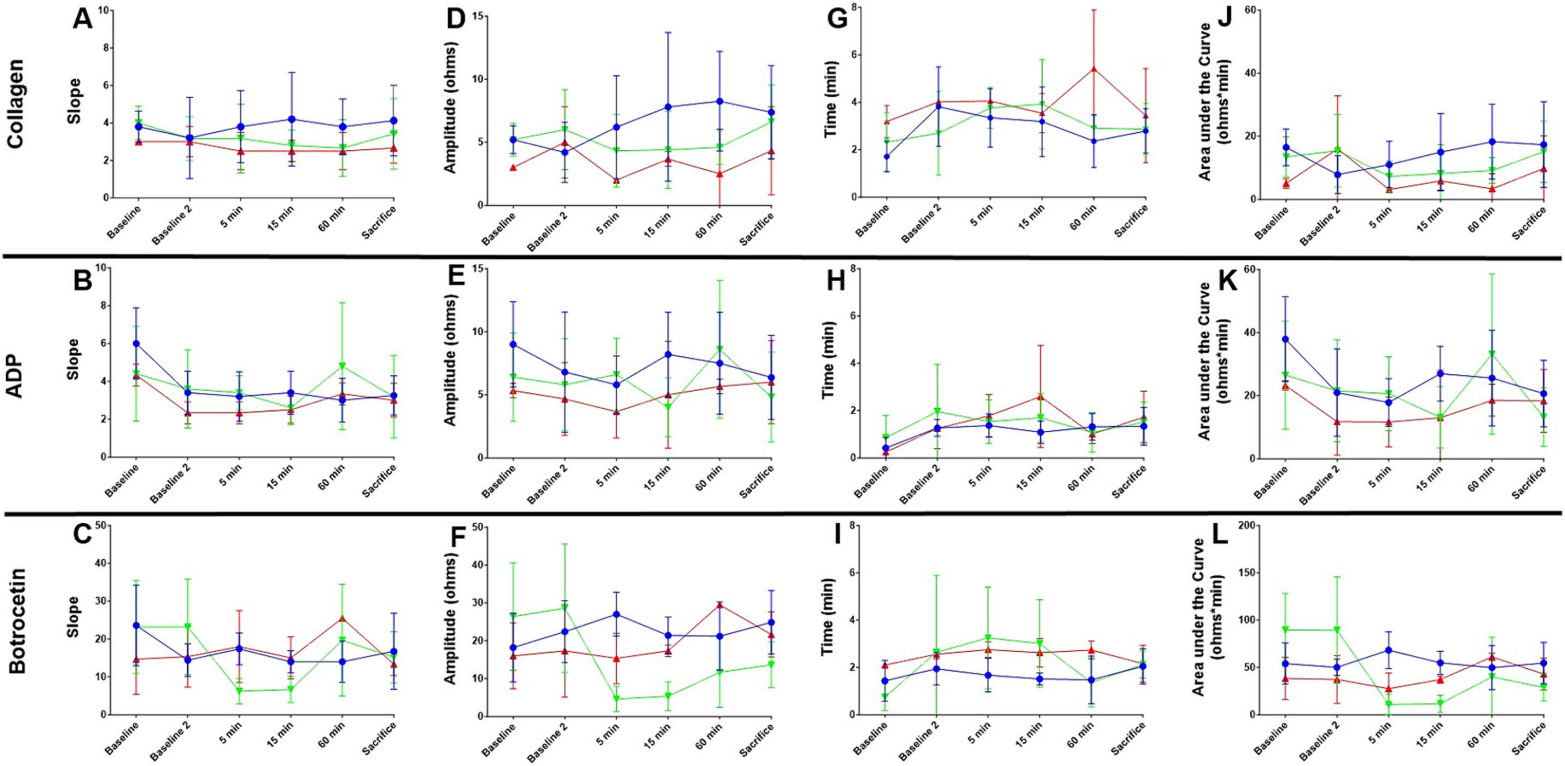
Whole blood impedance aggregometry in canine basilar artery occlusion. Slope **(A–C)**, Amplitude **(D–F)**, Lag time **(G–I)**, and area under the curve (AUC, **J–L**) are shown from baseline before catheter access manipulations, through 60 min of occlusion (baseline 2), 5, 15, and 60 min after treatment completion, and at sacrifice ~4 h after BAO. Values for vehicle (blue), rtPA (red), and BB-031 (green) are presented ± S.D. Statistical summary is presented in Table 2. Treatment with BB-031 demonstrated significantly less platelet activation and aggregation with botrocetin agonist compared to vehicle and rtPA.

Although the response to 3.2 μg/μL collagen was not significant with any treatment, there was a significantly lower amplitude at baseline in the canine group treated with 0.9 mg/kg rtPA (3.00 ± 0.00 ohms) compared to the vehicle (5.20 ± 1.1 ohms, *p* = 0.015) and 0.5 mg/kg BB-031 (5.21 ± 1.3 ohms, *p* = 0.029) (Figure 4). There was also a significantly shorter lag time at baseline with collagen in the canine group in the vehicle (1.71 ± 0.63 min) compared to 0.9 mg/kg rtPA (3.19 ± 0.68 min, *p* = 0.020). Lastly, in addition, a smaller collagen AUC resulted from rtPA administration (5.07 ± 1.53 ohms* min) compared to vehicle (16.48 ± 5.83 ohms* min, *p* = 0.018). None of these trends persisted 60 min after BAO before treatment was administered. Velocity is the maximal slope in the Chronolog recorded tracing after agonist introduction, and AUC reflects the overall platelet activity, which is affected by the height of the resulting reaction. Although slope and lag time were unremarkable with 20 μM ADP addition, a significant decrease was seen with BB-031 amplitude (4.00 ± 2.35 ohms) 15 min after treatment compared to the vehicle (8.25 ± 3.35 ohms, *p* = 0.050). In addition, a smaller AUC resulted from BB-031 administration (13.10 ± 9.74 ohms* min) 15 min after treatment compared to the vehicle (26.98 ± 8.68 ohms, *p* = 0.045).

##### Botrocetin agonist reactions

Botrocetin causes platelet aggregation through the VWF and GPIb-IX-V complex, and since we knew our mechanism of action was VWF inhibition, we expected to see major changes in botrocetin agonist action in canines that had been treated with BB-031 (summary of significance, Table 2). There was a significant decrease in *slope* in response to botrocetin at 5 min with BB-031 (6.20 ± 3.35) compared to vehicle (17.40 ± 4.22, *p* = 0.002) and compared to rtPA (18.01 ± 9.54, *p* = 0.039) replicated again at 15 min after BB-031 treatment (6.67 ± 3.44) compared to vehicle (14.00 ± 2.92, *p* = 0.004) and rtPA (15.02 ± 5.57, *p* = 0.025). The slope was also significantly lower with BB-031 (6.20 ± 3.35) compared to rtPA at 5 min (18.00 ± 9.54, *p* = 0.039). Interestingly, the rtPA group (25.50 ± 0.71) was significantly increased compared to vehicle at 60 min (14.00 ± 5.43, *p* = 0.039). BB-031 effect on *amplitude* was even stronger. Amplitude was significantly higher with vehicle (27.01 ± 5.79 ohms) compared to BB-031 at 5 min (4.60 ± 3.36 ohms, *p* < 0.0001) and compared to rtPA (15.33 ± 6.66 ohms, *p* < 0.021). BB-031 (5.33 ± 3.78 ohms) remained lower at 15 min than vehicle (21.40 ± 4.88 ohms, *p* < 0.001) and rtPA (17.33 ± 1.53 ohms, p = 0.025). This amplitude decrease continued with BB-031 at 60 min with BB-031 (11.67 ± 9.22 ohms) to that with rtPA (29.50 ± 0.71 ohms, *p* = 0.041) and at sacrifice in BB-031 (13.67 ± 6.07 ohms) compared to both rtPA (21.67 ± 5.99 ohms, *p* = 0.018) and vehicle (24.88 ± 8.36, *p* = 0.003). The only difference in *lag time* was seen at 15 min with BB-031 treatment (3.01 ± 1.85 min) which was significantly longer than vehicle (1.51 ± 0.25 min, *p* = 0.009). *AUC* at 5 min decreased with BB-031 (10.76 ± 11.00 ohms* min) compared to vehicle (68.20 ± 19.48 ohms* min, *p* < 0.0004), 15 min with BB-031 (11.70 ± 8.97 ohms* min) compared to vehicle (54.86 ± 12.22 ohms* min p < 0.0001), and at sacrifice with BB-031 (28.97 ± 14.36 ohms* min) compared to vehicle (54.64 ± 21.78 ohms* min, *p* = 0.005). Lastly, at 5 min after treatment, rtPA was decreased (27.57 ± 16.46 ohms* min) compared to vehicle (68.20 ± 19.48 ohms* min, *p* = 0.024). Although AUC for rtPA and vehicle had returned to baseline by time of sacrifice, BB-031 was still ~30% of the original baseline mean value.

**Table 2:** Whole blood response to botrocetin agonist.

Time Point	Vehicle (*n* = 8)	rtPA (*n* = 7)	BB-031 (*n* = 9)	*P-*value		
				Vehicle vs. rtPA	Vehicle vs. BB-031	rtPA vs. BB-031
Botrocetin slope						
Baseline	24 ± 11	15 ± 9	23 ± 12	0.276	0.957	0.343
Baseline 2	14 ± 4	15 ± 8	23 ± 13	0.834	0.179	0.378
5 min	17 ± 4	18 ± 10	6 ± 3	0.904	0.002	0.039
15 min	14 ± 3	15 ± 6	7 ± 3	0.744	0.004	0.025
60 min	14 ± 5	26 ± 1	20 ± 15	0.037	0.440	0.615
Sacrifice	17 ± 10	13 ± 3	15 ± 7	0.439	0.678	0.539
Botrocetin amplitude (ohms)						
Baseline	18 ± 9	16 ± 9	26 ± 14	0.748	0.309	0.304
Baseline 2	22 ± 8	17 ± 12	29 ± 17	0.501	0.483	0.360
5 min	27 ± 6	15 ± 7	5 ± 3	0.039	0.000	0.021
15 min	21 ± 5	17 ± 2	5 ± 4	0.221	0.000	0.001
60 min	21 ± 9	30 ± 1	12 ± 9	0.273	0.119	0.041
Sacrifice	25 ± 8	22 ± 6	14 ± 6	0.441	0.003	0.018
Botrocetin lag time (min)						
Baseline	1.43 ± 0.86	2.1 ± 0.11	0.74 ± 0.57	0.241	0.173	0.007
Baseline 2	1.94 ± 0.69	2.56 ± 0.20	2.65 ± 3.25	0.190	0.646	0.964
5 min	1.67 ± 0.71	2.75 ± 0.33	3.25 ± 2.16	0.052	0.159	0.713
15 min	1.51 ± 0.25	2.62 ± 0.60	3.01 ± 1.85	0.009	0.108	0.740
60 min	1.47 ± 1.0	2.74 ± 0.37	1.37 ± 1.03	0.156	0.875	0.129
Sacrifice	2.05 ± 0.75	2.16 ± 0.78	2.12 ± 0.57	0.794	0.815	0.903
Botrocetin AUC (ohms*min)						
Baseline	54.0 ± 21.7	38.3 ± 22.3	89.7 ± 38.6	0.362	0.110	0.084
Baseline 2	50.1 ± 8.7	37.3 ± 25.3	89.5 ± 56.2	0.323	0.161	0.189
5 min	68.2 ± 19.5	27.6 ± 16.5	10.8 ± 11.0	0.024	0.000	0.129
15 min	54.9 ± 12.2	37.1 ± 3.6	11.7 ± 9.0	0.055	0.000	0.002
60 min	49.8 ± 23.3	61.1 ± 3.7	40 ± 42.0	0.547	0.656	0.527
Sacrifice	54.6 ± 21.8	43 ± 16.8	29 ± 14.4	0.299	0.005	0.083

#### Recanalization efficiency

Of the 24 hounds entered into this study, thromboembolic BAO was successfully induced in all 24 dogs and confirmed by digital subtraction angiography. DSA was completed at baseline before occlusion, immediately before and after clot placement, 60 min after BAO, 60 min after treatment, and ~ 4 h after BAO (time of sacrifice) to determine location during access, thrombus size and location of the clot, and the extent of recanalization (Figure 1). Sixty minutes after treatment ended, canines were transported to the imaging suite, where magnetic resonance imaging (MRI) was completed. Animals returned to the angiography surgery suite within ~75 min. Laser speckle imaging began with baseline and was recorded continuously until MRI transport time, then continued again immediately before sacrifice (Figure 1).

##### Digital subtraction angiography and recanalization

**Figure 5:**
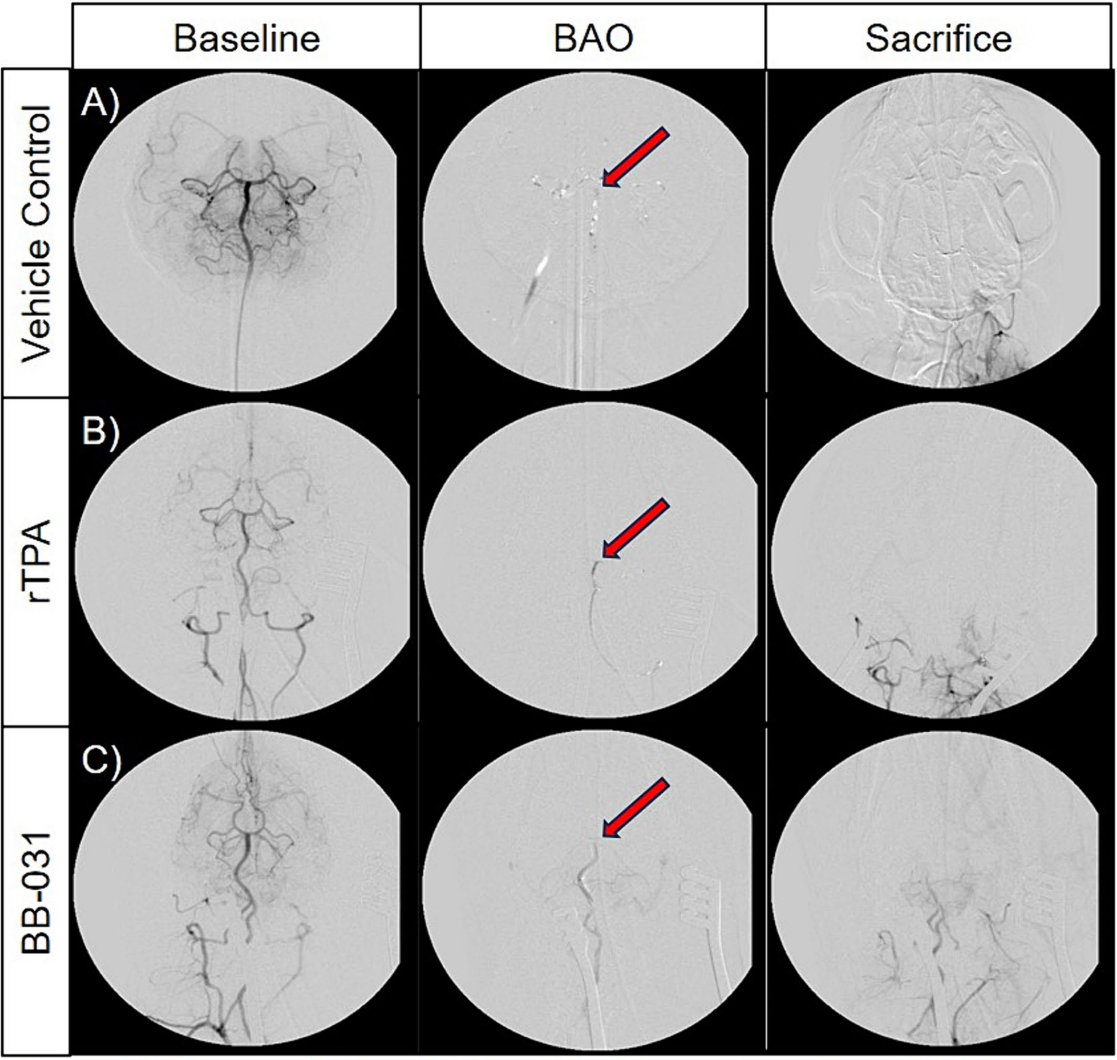
Representative digital subtraction angiography (DSA) images of basilar artery occlusion (BAO) in vehicle control canines **(A)**, 0.9 mg/kg rTPA treatment canines **(B)**, and 0.5 mg/kg BB-031 treatment canines **(C)** at baseline (row 1, TICI = 3), BAO (obtained 10-min post-injury, row 2, TICI = 0), and sacrifice (obtained ~4 h after occlusion, row 3). BAO site of clot placement is indicated by the red arrow in each representative images. The BB-031 treatment (TICI = 1–2) cohort displayed greater reperfusion of posterior cerebral circulation when compared to the vehicle control (TICI = 0) and rtPA (TICI = 0–1) cohorts. TICI Grade 3 = complete perfusion, 2 = partial or complete slower filling, 1 = minimal perfusion, 0 = no perfusion of occluded vessel or vessel territory.

After baseline DSA was established (Figures 5A–C), consistent and sustained BAO was sustained in all 24 hounds after clot placement with no evidence of microembolism through time of sacrifice(Figures 5D–F). We found no evidence of spontaneous recanalization or intracranial perforation through serial digital subtraction angiograms during the access procedures or before/after transport for MR imaging. Although none of the vehicle treated canines resulted in TICI scoring improvement after baseline, one canine treated with 0.9 mg/kg rtPA did achieve a TICI score of 1 immediately before sacrifice (Figures G–I). BB-031 administration resulted in ≥TICI 2A in 11% and ≥ TICI 1 in 55.5% of canines compared to rtPA (*p* = 0.042). Perfusion was also significantly increased compared to vehicle (TICI 0, *p* < 0.005).

##### Laser speckle imaging

**Figure 6:**
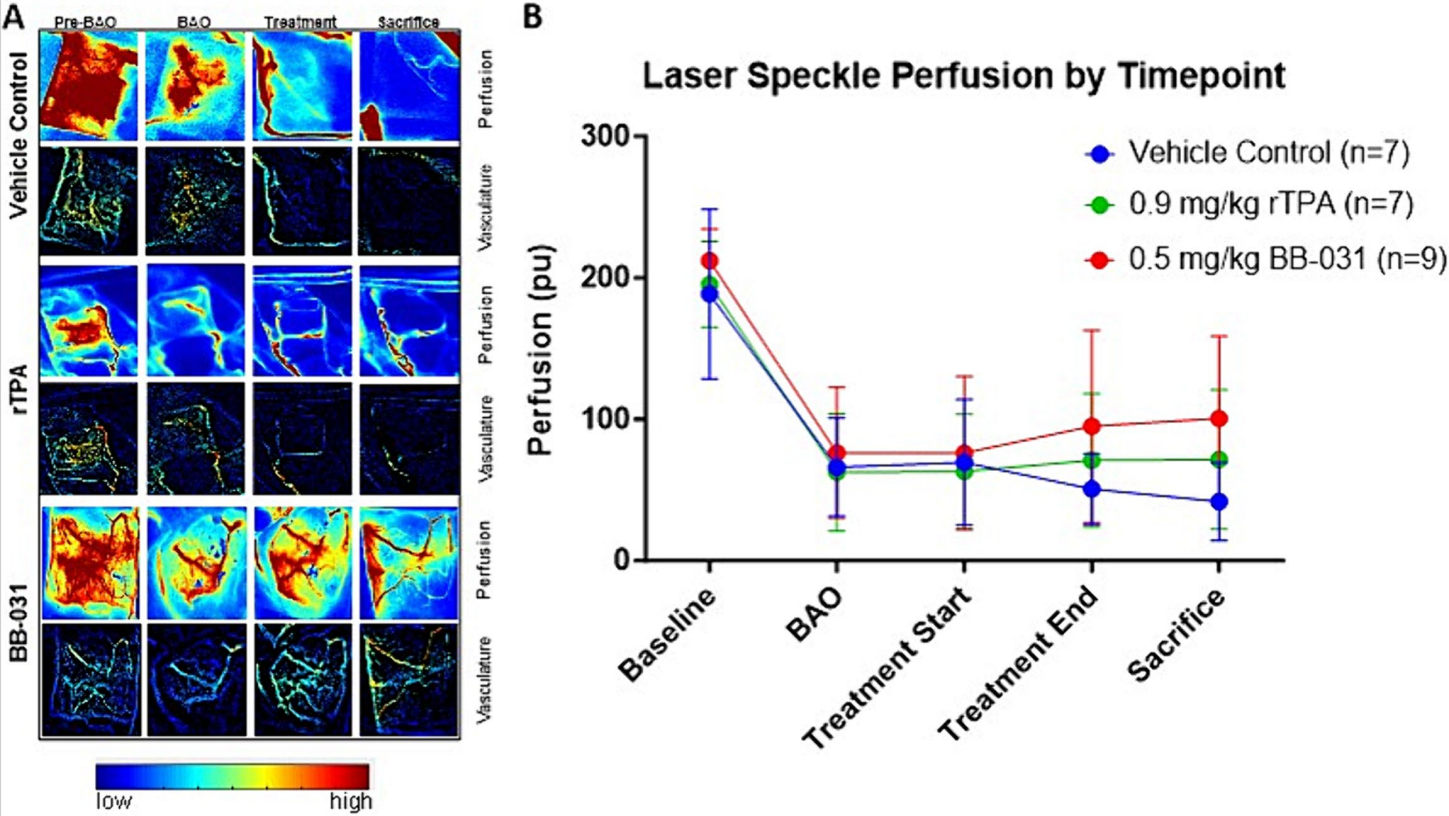
Representative laser speckle imaging (LSI). **(A)** Shows LSI at baseline, at BAO, treatment start, treatment end, and sacrifice through the cranial window. High flow is measured in red while low flow is blue. **(B)** BB-031 treated canines demonstrated improved perfusion compared to vehicle control at the sacrifice time point. (*p* = 0.028). [Abbreviations: basilar artery occlusion (BAO), recombinant tissue plasminogen activator (rtPA)].

Perfusion recording was performed continuously until the animals were transported to MRI, and again recorded immediately before sacrifice (Figure 6). In concordance with the angiograms, laser speckle perfusion imaging observed through the cranial window depicted unrestricted perfusion in the basilar artery prior to BAO (Figure 6A, first column) and fully occluded after administration of the autologous clot (Figure 6A, second column). Injection of the prepared thromboembolus dropped post BAO perfusion to a mean of 65 ± 35% in the vehicle group, 68 ± 42% in the rtPA treated group, and 64 ± 29% in the BB-031 treated group, representing an average decline of ~66% from baseline. The efficacy of recanalization in the BAO model was then verified (Figure 6A, third column). Although treatment with BB-031 was the only group that resulted in increased perfusion after treatment, it was not significantly different than vehicle or rtPA until sacrifice (Figure 6A, last column). At sacrifice, BB-031 had increased perfusion to 101 ± 58 pu compared to vehicle (42 ± 28 pu, *p* = 0.028), which had further decreased 37%, whereas rtPA (72 ± 69), *p* = 0.308 had only increased marginally (Figure 6B). In summary, BB-031 returned perfusion through the cranial window to a mean of 47% of its original baseline compared to rtPA at 37% and vehicle at 22% of their original baselines, respectively.

#### Infarct size reduction

##### Magnetic resonance imaging

**Figure 7:**
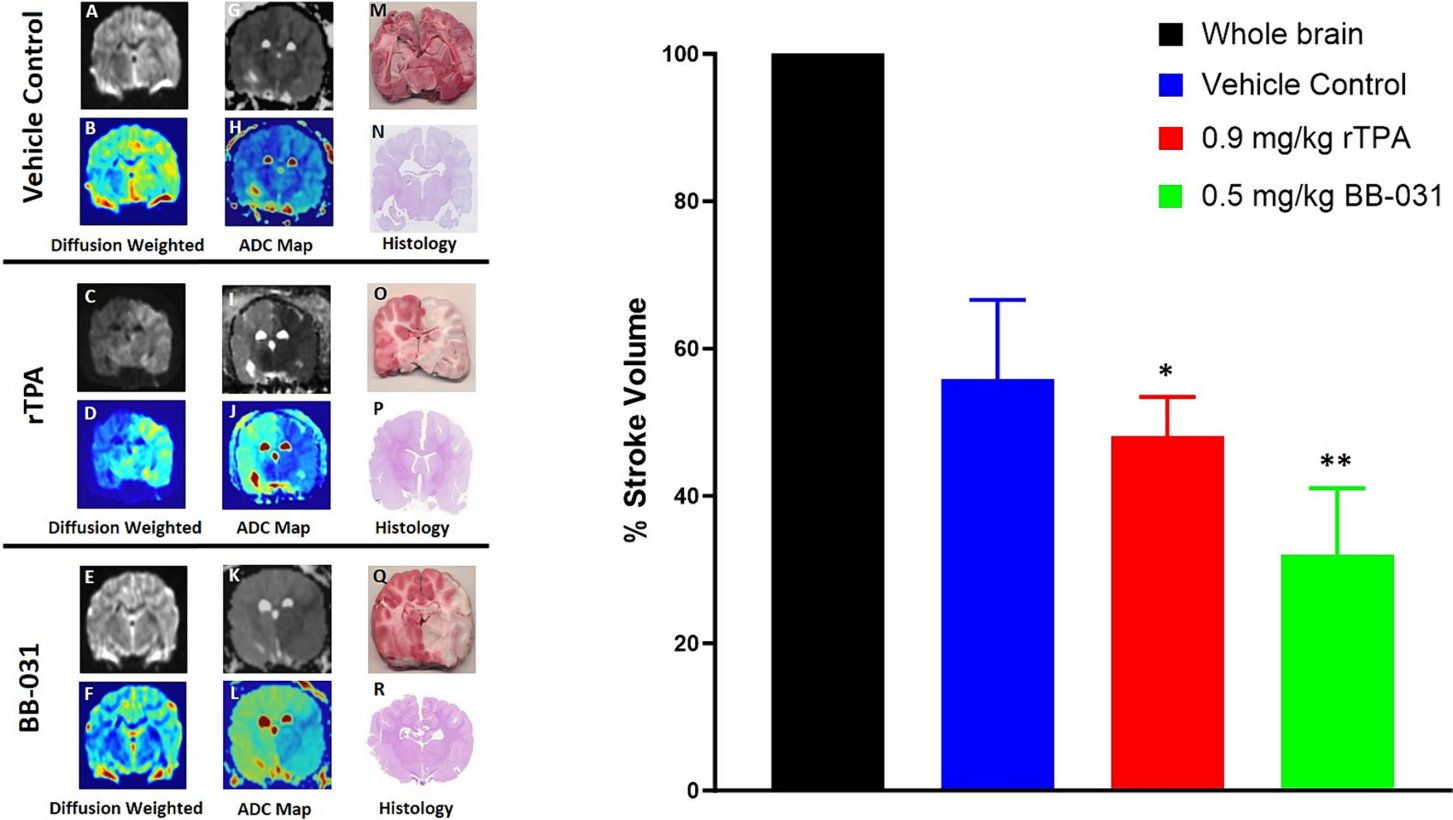
Representative magnetic resonance imaging (MRI). Diffusion weighted imaging (DWI) in grayscale in representative vehicle **(A)**, 0.9 mg/kg rtPA **(C)**, and 0.5 mg/kg BB-031 **(E)** and corresponding pseudocolor **(B,D,F)** showing hyperintense BA territory infarct. ADC maps in grayscale in representative vehicle **(G)**, 0.9 mg/kg rtPA **(I)**, and 0.5 mg/kg BB-031 **(K)** and corresponding pseudocolor **(H,J,L)** showing hypointense BA territory infarct. 2,3,5-triphenyl-2H-tetrazolium chloride (TTC) stained coronal brain section identifying areas in red that are still metabolically active compared to white inactive regions after BAO **(M,O,Q)** and hematoxylin and eosin (H&E) stained coronal brain section **(N,P,R)** confirmed infarct size and location. Graph inset summarizes the mean infarct volume across treatments. Treatment with BB-031 demonstrated significantly less infarct compared to vehicle and rTPA (*p* = 0.0026 and *p* = 0.0319, respectively).

Diffusion-weighted imaging (DWI) is the most sensitive imaging modality to detect acute infarcts, and high b-value apparent diffusion coefficient (ADC) mapping was performed to enable quantification of acute ischemic stroke volume (35). Data was converted to DICOM images and transferred for post-processing. The ideal b-value in adult humans is *b* = 1,000 s/mm^2^, in neonates it is b = 600 s/mm^2^. In this canine model, we found the ideal DWI at *b* = 1,800 s/mm^2^ (27). Therefore, DW images corresponding to the b-values of 0 and 1,800 s/mm^2^ of vehicle treated canines (Figures 7A,B), rtPA treated canines (Figures 7C,D), and BB-031 treated canines (Figures 7E,F) were used to obtain ADC maps. Hyperintense regions on DWI represent infarction corresponding to hypointense region on ADC and corresponding pseudocolor images vehicle treated canines (Figures 7G,H), rtPA treated canines (Figures 7I,J), and BB-031 treated canines (Figures 7K,L). The infarct region, traced throughout with red dotted lines, correlated to TTC (Figures 7M,O,Q) and H&E (Figures 7N,P,R) staining in the same brain slice. Quantification of infarct volume was performed by delineating the lesion borders on DICOM images using OsiriX MD v.5.0 software to calculate the areas. Area of the whole brain and infarct region so obtained were multiplied by the slice thickness (3 mm) to get the brain and infarct volumes. Whole brain volume was converted as 100 percent and the infarct volume percent was calculated. Data = mean ± SD. Percent stroke volume with 0.5 mg/kg BB-031(31.96 ± 9.10) was significantly smaller compared to vehicle (55.82 ± 10.78, *p* = 0.0026) and rtPA (48.06 ± 5.37, *p* = 0.0319). Hemorrhage was identified in 6/7 canines treated with rtPA. Hemorrhage, located in the right occipital was identified in only one of the canines at this early sacrifice time point.

## Discussion

Our results unequivocally provide evidence that VWF aptamer BB-031 is an effective thrombolytic agent in a large animal model of posterior circulation stroke. Compared to vehicle and clinical regime rtPA, BB-031 provided superior recanalization of the basilar artery and surrounding territory without evidence of hemorrhage risk. BB-031 bolus resulted in anti-platelet effects through botrocetin agonist action throughout the treatment period, which lasted 40 min and was shorter than rtPA continuous infusion. BB-031 was administered as an injection over 5 min while the rtPA was infused over 45 min. This slow infusion of rtPA is necessary in humans, as more rapid infusion results in higher rates of hemorrhage with no improvement in recanalization (34). This effect persisted for more than 140 min after drug administration until the experiment was terminated. As the VWF aptamer inhibits platelet recruitment and activation, PAI-1 levels may be reduced in thrombosis in the aptamer-treated canines, which may, in part, result in improved recanalization rates in arterial platelet-rich clots with AIS. BB-031 resulted in decreased infarct size, returned blood flow throughout the BAO territory, and reduced platelet aggregation and activation while maintaining blood homeostasis as provided by evidence of normal platelet activation with collagen and ADP in two separate assays after acute BAO compared to vehicle and most importantly, rtPA.

We expected changes in circulating VWF and ADAMTS13 but were pleasantly surprised that platelet effects extended to the sacrifice time ~ 4 h after BAO. Matrix metalloproteinase 9 (MMP-9), known to digest basal lamina, contributing to blood–brain barrier (BBB) damage, decreased in both rtPA and BB-031 at sacrifice but did not reach significance. Although another group showed circulating MMP-9 was significantly increased with 2 mg/kg rtPA in an autologous clot-induced MCAO canine model, levels returned to MCAO without rtPA within 2 h of administration (27). Significance in this model was not reached with MMP-9 until 5.5 h of initiation; therefore, we knew our sacrifice time was outside the significant increase if canine BAO is comparable to MCAO. We measured MMP-9 because, to our knowledge, the protein has not been characterized in the BAO canine model. Future studies utilizing B0-031 in a canine model of MCAO will look at MMP-9 levels from baseline through 9 h of ischemia to compare with a time point outside the therapeutic window of rtPA.

There are several *limitations* in our study. First, although our pre-programmed MRI imaging software scans for the time of flight magnetic resonance angiography (TOF-MRA), T2-weighted, and FLAIR imaging, we did not include them in our figures as we demonstrated when we characterized this model of acute basilar artery occlusion that these two imaging modalities are inadequate to detect early acute strokes (30). Our optimized b-value DWI for the dog detected the acute ischemic lesion within 4 h post-BAO. An optimized b-value is essential to achieve ideal sensitivity and differs depending on what is being studied. ADC maps corresponding to high b-value are more sensitive for hyper-acute ischemic stroke as early as 30 min after stroke onset (36). Furthermore, the ADC maps enabled better discrimination of infarct and normal tissue, thereby increasing the accuracy of tracing lesion borders for accurate infarct volume calculation (37).

Our large animal stroke model of choice was canines. Canines have a gyrencephalic brain, which is closer to humans, comparing white to gray matter, than small animals, and sheep and swine have rete mirable that supplies the cerebral arteries not evident in humans or dogs (38). We also placed the autologous clot in the middle of the BA to recapitulate clinical BAO (3, 39). Our investigation of the efficacy of BB-031 in large animal stroke began in the basilar artery as opposed to the MCA, where anterior strokes commonly occur. This was our intention as we feel the lack of data collected in posterior circulation strokes makes many clinical trials that study thrombolytics inadequate as few patients with BAO are being enrolled and critical recanalization in BAO addressed. A TICI score of 2b is defined as adequate reperfusion, and only 2 of our BB-031 treated canines reached that score. This is a severe nonsurvival canine basilar injury because none of our canines could breathe on their own during MRI transport, and our time of sacrifice was short (no time for improvement as progression of recovery is slow in BAO). Therefore, no behavioral assessments could be provided with this model, but it replicates the severity of clinical BAO. Furthermore, NIHSS scores might not accurately reflect clinical severity in posterior circulation stroke, and score frequently unavailable for patients with BAO who arrive at the ER intubated (6).

VWF did not decrease at every time point we sampled with BB-031 administration. Still, we were limited by the number of whole blood draws that could be performed without affecting the physiological parameters of the surgery and platelet function. Future studies in the MCAO model planned will utilize hounds so that the extra weight (~3.5 times > beagles) can be used to characterize better BB-031 circulating effect on VWF, ADAMTS13, and MMP-9 and, just as significantly, beyond the therapeutic window afforded by rtPA at 4.5 h. We would have also liked to utilize equal numbers of male and female beagles to tease out these differences better, but we were limited by sex and availability at the time of the study. Although stroke differences occur with sex, with females having more severe anterior strokes and more significant disability, males have more frequent posterior circulation strokes and are younger at the time of onset (39, 40). Most BAO patients are <50 years of age, but we do not use aged canines due to the availability of aged canines for research purposes. Small animal numbers are expected, with an average of 6 per group in canine stroke studies (33). However, the necessity of canines compared to ovine and porcine due to anatomy and an autologous clot model with MRI/DSA is more appropriate for thrombolytic studies.

Some studies showed that pharmacological agents with intra-arterial treatment (IAT) resulted in higher recanalization compared to intravenous treatment (IVT) but with a consequent increase in hemorrhage and poor outcome with IAT in moderate severity strokes. We did not evaluate IAT with BB-031. Qureshi 2004 studied IAT vs. IVT in BAO dogs and found recanalization rates similar, but hemorrhage rates higher with IVT (28). Rate of recanalization with rtPA was comparable with this group as they arrived at 1/6 with IVT whereas we did not identify any reperfusion in rtPA-treated canines. This difference may be explained by the difference in treatment time after BAO (1 h vs. 2) and their reported recanalization measure was partial in description. In their extended monitoring time of 6 h, that observed recanalization disappeared. IVT alone with current treatments fails to recanalize in thrombosis exceeding 13 mm in BAO (4). BB-031 IAT administration is a planned future study (1, 5). Unfortunately, neither large animal study can afford insight into long term treatment or ischemic effects as the severity of the ischemic location has not allowed survival studies. During transport to imaging before sacrifice, our animals could not be extubated and controlled ventilation was necessary. This inevitably limits animal studies to draw from, particularly in large animals. Lastly, in an elegant study by Denorme in 2021, the permanent murine MCAO model was used to demonstrate that exacerbation of inflammation due to VWF increase is centered on the A1 domain, making BB-031 inhibition of VWF an optimal AIS treatment agent (41).

In summary, although not the focus of this investigation, we developed an aptamer-based antidote to BB-031, termed BB-025, which we have shown rapidly reverses BB-031 activity. Posterior stroke is associated with higher risk of recurrent stroke (2). Future large animal studies have translated BB-031 thrombolytic efficacy to MCAO and characterized the kinetics and dose–response of BB-025 after ischemia and BB-031 thrombolysis. Both our canine BAO and MCAO investigations have laid the foundation for Phase I Clinical Trials which provided safety data in healthy controls for progress to Phase II which will begin as this manuscript goes to publication.

## Statistical analysis

Values are expressed as mean ± SD. Statistical analysis was performed using multiple t tests to compare vehicle with treatment groups in physiological baseline and sacrifice parameters, MMP-9 levels, and stroke volumes. Two-way ANOVA was used to compare BB-031, rtPA, and vehicle control in the canine model of basilar artery vessel occlusion to compare platelet inhibition of BB-031 in canine whole blood and VWF/ADAMTS13 levels throughout the procedures. Kolmogorov–Smirnov test (KS test) was used to compare hemorrhage.

## Data Availability

The raw data supporting the conclusions of this article will be made available by the authors, without undue reservation.
